# Description of a new species of the leafhopper genus *Zyginella* Löw from Southwest China (Hemiptera, Cicadellidae, Typhlocybinae)

**DOI:** 10.3897/zookeys.168.2171

**Published:** 2012-01-31

**Authors:** Yuehua Song, Zizhong Li

**Affiliations:** 1 Institute of Entomology, Guizhou University, Guiyang, Guizhou 550025, China; 2 Institute of South China Karst, Guizhou Normal University, Guiyang, Guizhou 550001, China; 3 The State Key Laboratory Incubation Base for Karst Mountain Ecology Environment of Guizhou Province, Guiyang, Guizhou 550001, China

**Keywords:** Morphology, taxonomy

## Abstract

A new species, *Zyginella menghaiensis* **sp. n.** (Hemiptera: Cicadellidae: Typhlocybinae: Zyginellini), is described from Chinaand a key to species of *Zyginella* from China is provided.

## Introduction

The leafhopper genus *Zyginella* was established by Löw in 1885. The genus belongs in the tribe Zyginellini of Typhlocybinae and consists of twenty-two species distributed in the Oriental, Palaearctic and Afrotropical Regions. Members of the genus can be distinguished by the distinct dark spot on the 3rd apical cell of the forewing (Fig. 3) and in the male genitalia by the male pygofer with short ventral caudal process and long macrosetae on the posterodorsal margin (Fig. 5) and style elongate, slender throughout length with truncate base (Fig. 7).

Recent taxonomic work on the genus includes Dworakowska (1969, 1970, 1974, 1977), Chiang et al. (1988) and Zhang (1990); up to now, eight species of *Zyginella* have been recorded from China in these studies. In the current work, a new species from Yunnan Province, China is described and illustrated and a key to Chinese species of *Zyginella* is given. All specimens examined are deposited to the collection of the Insititute of Entomology, Guizhou University, Guiyang, China (GUGC).

## Taxonomy

### Zyginella

Löw

http://species-id.net/wiki/Zyginella

Zyginella Löw, 1885: 346; Dworakowska 1969: 433, 1970: 707, 1974: 161, 1977: 24; Chiang et al. 1988: 109; Zhang 1990: 170. Type species: *Zyginella pulchra* Löw, 1885.
Pyramidotettix Matsumura, 1932: 59; Dworakowska 1970: 707; Chiang et al. 1988: 109; Yang 1965: 197. Type species: *Conometopius citri* Matsumura, 1907. Synonymized by Dworakowska 1970: 707.
Remmia Vilbaste, 1968: 91; Dworakowska 1970: 707; Chiang et al. 1988: 109. Type species: *Remmia orbigera* Vilbaste, 1968. Synonymized by Dworakowska 1970: 707.

#### Description.

 Forewing (Fig. 3) with distinct dark spot on 3rd apical cell.

**Figures 1–13.**
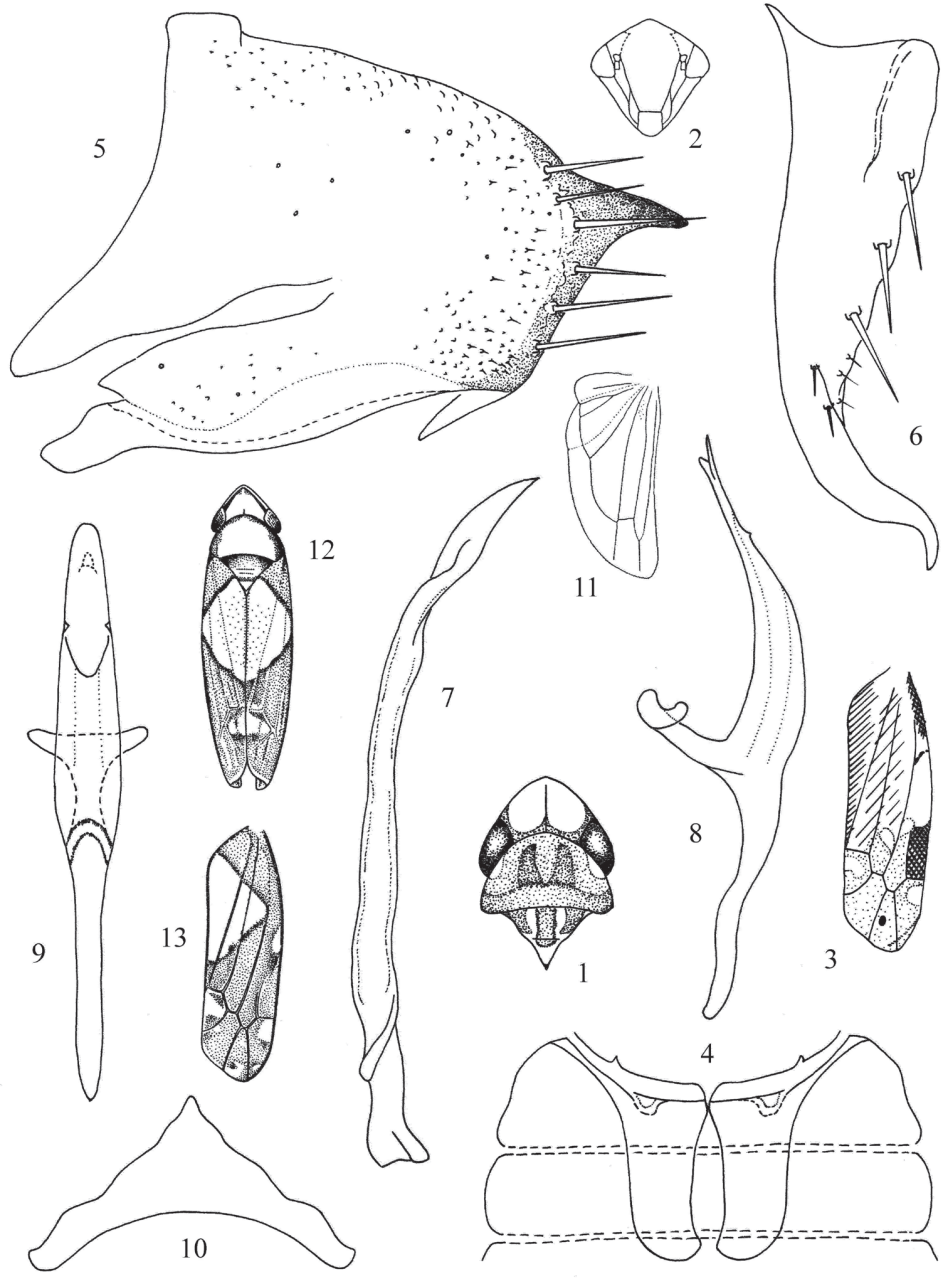
*Zyginella* species **1–11** *Zyginella menghaiensis* sp. n. **1** Head and thorax, dorsal view **2** Face **3** Forewing **4** Abdominal apodemes **5** Pygofer lobe, lateral view **6** Subgenital plate **7** Style **8** Aedeagus, lateral view **9** Aedeagus, ventral view **10** Connective **11** Hindwing **12–13** *Zyginella minuta* (after Yang, 1965) **12** Adult, dorsal view **13** Forewing.

Head (Fig. 1) acutely produced medially, about as wide as greatest width of pronotum; coronal suture prominent. Forewing (Fig. 3) with 1st apical cell short. Hind wing (Fig. 11) with submarginal vein confluent with Cu_1_ markedly distad of point of fusion of Cu_1_ with M_3+4_.

Male pygofer (Fig. 5) with short process on lateroventral margin and numerous long macrosetae on posterodorsal surface. Subgenital plate usually forming a pocket-like structure at tip or tapering towards apex (Fig. 6). Style (Fig. 7) broadened and truncate at base. Aedeagal shaft (Figs 8, 9) usually curved dorsally; preatrium long or short; dorsal apodeme narrow. Connective (Fig. 10) V- or Y-shaped; lateral arms long; central lobe absent.

#### Distribution.

 Oriental region, Palaearctic region, Afrotropical region.

#### Key to Chinese species of the genus Zyginella

**(males only couplets 5–7)**

**Table:** 

1	Vertex with black stripe between eyes subapically (Fig. 12)	2
–	Vertex without black stripe between eyes subapically (Fig. 1)	4
2	Pronotum with two black transverse stripes	*Zyginella citri* (Matsumura)
–	Pronotum without black transverse stripes	3
3	Vertex and pronotum with pair of dark spots respectively	*Zyginella mali* (Yang)
–	Vertex and pronotum without spots	*Zyginella minuta* (Yang)
4	Forewings without a large rhombus-like patch along inner margin subbasally (Fig. 3)	5
–	Forewings with a large rhombus-like patch along inner margin subbasally (Figs 12, 13)	7
5	Aedeagus preatrium well developed, about as long as length of aedeagal shaft (Figs 8, 9)	6
–	Aedeagus preatrium vestigial	*Zyginella orla* Dworakowska
6	Aedeagal shaft with a single short dorsal process subapically (Figs 8, 9)	*Zyginella menghaiensis*sp. n.
–	Aedeagal shaft without a single short dorsal processes subapically	*Zyginella tsauri* Chiang, Hsu & Knight
7	Pygofer lobe with a hook-like process caudo-dorsally	*Zyginella punctata* Zhang
–	Pygofer lobe without hook-like process caudo-dorsally	*Zyginella taiwana* Chiang, Lee & Knight

### Zyginella menghaiensis

Song & Li sp. n.

urn:lsid:zoobank.org:act:090D3B2C-B357-4E11-9F81-AC71C63C6743

http://species-id.net/wiki/Zyginella_menghaiensis

Figures 1–11

#### Description.

 Head and thorax yellowish brown; vertex with lateral margins with soft red tinge; eyes brownish grey; pronotum brownish with two longitudinal darker stripes; scutellum with basal triangles testaceous. Forewing (Fig. 3) reddish brown near base, dark red between 4th apical cell and brochosome-field and light brown around apex; 3rd apical cell with a blackish brown spot.

Coronal suture (Fig. 1) extending nearly to anterior margin of vertex. Forewing (Fig. 3) with 3rd apical cell not petiolate at base.

Abdominal apodemes (Fig. 4) slender, slightly extended beyond 4th sternite.

Pygofer lobe (Fig. 5) broad, with a large sclerotized process near dorsal margin and another process arising from about ventro-caudal margin; six long macrosetae distributed along caudal margin and numerous short microsetae scattered on lateral surface. Subgenital plate (Fig. 6) long, gradually tapered towards apex and curved apically, beak-like; with three long macrosetae along upper margin. Style (Fig. 7) elongate, slender throughout length with truncate base. Aedeagal shaft (Figs 8, 9) curved dorsad in lateral view with single small dorsal process subapically; gonopore large, apical on ventral surface with small tooth on each lateral margin; preatrium long, about as long as aedeagal shaft; dorsal apodeme narrow. Connective (Fig. 10) Y-shaped with very short stem and long strongly divergent lateral arms; central lobe absent.

#### Measurement.

 Body length males 2.9~3.1 mm.

#### Type material.

*Holotype*, male, China: Yunnan Province, Menghai County, 23 July 2008, coll. YUE-HUA SONG. *Paratypes*: two males, same date as holotype.

#### Remarks.

 The new species is similar to *Zyginella tsauri* Chiang, Hsu & Knight (1989), but the forewing has a large dark costal patch (Fig. 3) and the aedeagus has a single short dorsal process subapically and a small tooth on each lateral margin of the gonopore (Figs 8, 9).

#### Etymology.

 The new species is named for its type locality: Menghai.

## Supplementary Material

XML Treatment for Zyginella

XML Treatment for Zyginella menghaiensis
